# Supplementary material for: Safety and Immunogenicity of a 23-Valent Pneumococcal Polysaccharide Vaccine (PPSV23) in Chinese Children, Adults and the Elderly: A Phase 4, Randomized, Double-Blind, Active-Controlled Clinical Trial
Source: Vaccines (Basel). 2025 Aug 15;13(8):866. doi: 10.3390/vaccines13080866 (PMC12389771; doi:10.3390/vaccines13080866)
Supplement: Supplementary file 1 [file vaccines-13-00866-s001.zip › vaccines-3770935-supplementary.pdf]

Table S1. Serological Response of study participants aged 2–17 years to the 23-valent Pneumococcal Polysaccharide Vaccines.

Serotypes	GMC (95%CI) at baseline		*p*-*value*	GMC (95%CI) post-vaccination		GMC ratio (95%CI) post-vaccination	*p*-*value*	Seroconversion rate (95%CI)		*p*-*value*
	Treatment (*n* = 587)	Control (*n* = 295)		Treatment (*n* = 587)	Control (*n* = 295)			Treatment (*n* = 587)	Control (*n* = 295)	
3	0.36(0.31–0.41)	0.38(0.32–0.46)	0.5082	1.34(1.26–1.43)	1.57(1.44–1.72)	0.85(0.76–0.95)	**0.0042**	57.58(53.47–61.62)	63.73(57.96–69.22)	0.0791
6B	1.63(1.52–1.74)	1.67(1.52–1.85)	0.6374	5.25(4.79–5.74)	4.89(4.30–5.56)	1.07(0.92–1.25)	0.3841	66.78(62.81–70.58)	58.64(52.79–64.32)	**0.0175**
14	5.60(5.26–5.98)	5.68(5.18–6.21)	0.8232	32.51(29.41–35.93)	29.52(25.63–34.00)	1.10(0.93–1.31)	0.2745	80.24(76.78–83.39)	78.64(73.52–83.18)	0.5786
19F	2.90(2.72–3.09)	2.96(2.71–3.24)	0.684	12.28(11.34–13.30)	12.00(10.72–13.43)	1.02(0.89–1.18)	0.7374	79.05(75.53–82.27)	76.61(71.36–81.32)	0.4082
19A	3.15(2.94–3.37)	3.29(2.99–3.63)	0.4572	10.19(9.43–11.01)	10.39(9.32–11.59)	0.98(0.86–1.12)	0.7717	66.95(62.98–70.75)	66.44(60.74–71.81)	0.8794
23F	1.14(1.07–1.22)	1.18(1.07–1.30)	0.6006	5.15(4.70–5.64)	4.17(3.67–4.73)	1.24(1.06–1.45)	**0.0079**	77.17(73.56–80.51)	69.49(63.89–74.70)	**0.0134**
1	0.42(0.38–0.46)	0.44(0.38–0.50)	0.5938	4.47(4.15–4.81)	4.36(3.93–4.84)	1.02(0.90–1.16)	0.717	96.76(94.99–98.04)	93.22(89.72–95.81)	**0.0157**
2	2.41(2.27–2.56)	2.50(2.29–2.72)	0.5134	22.09(20.69–23.59)	18.44(16.81–20.23)	1.20(1.07–1.34)	**0.0018**	96.42(94.58–97.77)	92.88(89.32–95.54)	**0.0198**
4	0.24(0.21–0.28)	0.25(0.21–0.30)	0.8627	3.97(3.72–4.24)	2.93(2.68–3.21)	1.36(1.21–1.51)	**<0.0001**	94.89(92.78–96.53)	89.49(85.42–92.75)	**0.0029**
5	0.41(0.37–0.45)	0.41(0.36–0.47)	0.9332	3.69(3.43–3.97)	3.27(2.95–3.63)	1.13(0.99–1.28)	0.0634	95.91(93.98–97.36)	94.24(90.93–96.61)	0.2652
7F	0.75(0.69–0.83)	0.79(0.69–0.90)	0.5579	5.82(5.40–6.28)	5.40(4.86–6.01)	1.08(0.95–1.23)	0.2646	93.53(91.22–95.38)	90.85(86.96–93.88)	0.1508
8	1.57(1.44–1.71)	1.60(1.42–1.81)	0.7772	22.39(21.05–23.81)	18.35(16.83–20.01)	1.22(1.10–1.36)	**0.0003**	96.25(94.38–97.64)	94.92(91.75–97.13)	0.3501
9N	0.55(0.48–0.64)	0.60(0.49–0.74)	0.5102	8.31(7.75–8.92)	7.45(6.75–8.23)	1.12(0.99–1.26)	0.0783	89.78(87.04–92.11)	86.44(82.00–90.13)	0.1402
9V	0.90(0.81–1.00)	0.92(0.79–1.07)	0.8154	4.84(4.49–5.23)	4.27(3.83–4.75)	1.14(1.00–1.30)	0.0575	86.37(83.33–89.04)	81.02(76.07–85.33)	**0.0378**
10A	2.21(2.09–2.33)	2.28(2.11–2.46)	0.4889	6.79(6.29–7.33)	7.07(6.35–7.88)	0.96(0.84–1.10)	0.5476	64.40(60.37–68.27)	64.41(58.65–69.87)	0.9973
11A	1.21(1.08–1.36)	1.47(1.25–1.72)	0.0605	5.06(4.75–5.38)	4.49(4.11–4.90)	1.13(1.01–1.25)	**0.029**	71.38(67.54–75.01)	61.69(55.88–67.27)	**0.0036**
12F	0.22(0.20–0.24)	0.24(0.21–0.28)	0.1562	1.64(1.53–1.76)	1.60(1.45–1.77)	1.02(0.90–1.15)	0.7468	91.48(88.92–93.61)	91.19(87.35–94.16)	0.8826
15B	2.12(1.94–2.31)	2.22(1.96–2.52)	0.5265	16.59(15.02–18.32)	13.4(11.65–15.42)	1.24(1.04–1.47)	**0.0149**	90.46(87.79–92.71)	84.75(80.13–88.65)	**0.0119**
17F	0.75(0.69–0.82)	0.81(0.72–0.92)	0.3423	6.20(5.81–6.62)	5.98(5.45–6.55)	1.04(0.93–1.16)	0.5173	94.38(92.20–96.10)	92.20(88.53–94.99)	0.2114
18C	0.73(0.64–0.82)	0.82(0.69–0.97)	0.2463	5.98(5.54–6.45)	5.32(4.78–5.92)	1.12(0.99–1.28)	0.0805	91.48(88.92–93.61)	88.14(83.89–91.60)	0.1121
20	2.05(1.88–2.23)	2.06(1.83–2.33)	0.9057	6.72(6.32–7.14)	6.44(5.90–7.02)	1.04(0.94–1.16)	0.4383	70.02(66.13–73.70)	61.02(55.19–66.62)	**0.0073**
22F	1.79(1.70–1.89)	1.86(1.73–2.01)	0.4246	8.44(7.88–9.04)	8.87(8.06–9.77)	0.95(0.85–1.07)	0.4059	80.75(77.32–83.86)	84.07(79.38–88.05)	0.2276
33F	0.40(0.35–0.46)	0.46(0.38–0.56)	0.2756	8.59(7.98–9.25)	7.41(6.68–8.22)	1.16(1.02–1.32)	**0.0222**	97.79(96.24–98.82)	98.31(96.09–99.45)	0.6065

Note: The values in parentheses represent 95% confidence intervals, formatted as (lower bound–upper bound). GMC= geometric mean concentration; GMI= geometric mean fold increase; 95%CI= 95% confidence interval.

Table S2. Serological Response of study participants aged 18–59 years to the 23-valent Pneumococcal Polysaccharide Vaccines.

Sero-types	GMC (95%CI) at baseline		*p*-value	GMC (95%CI) post-vaccination		GMC ratio (95%CI) post-vaccination	*p*-value	Seroconversion rate (95%CI)		*p*-value
	Treatment (*n* = 235)	Control (*n* = 117)		Treatment (*n* = 235)	Control (*n* = 117)			Treatment (*n* = 235)	Control (*n* = 117)	
3	0.32(0.29–0.36)	0.33(0.28–0.39)	0.8278	0.81(0.73–0.89)	0.88(0.77–1.01)	0.92(0.78–1.09)	0.3386	64.68(58.20–70.78)	61.54(52.09–70.38)	0.5637
6B	2.41(2.18–2.66)	2.11(1.83–2.43)	0.1294	8.55(7.48–9.76)	7.02(5.81–8.48)	1.22(0.97–1.54)	0.0945	72.34(66.15–77.96)	68.38(59.13–76.66)	0.4399
14	8.31(7.58–9.11)	8.70(7.64–9.91)	0.578	32.20(28.25–36.70)	31.82(26.43–38.30)	1.01(0.81–1.27)	0.9173	76.17(70.20–81.47)	71.79(62.73–79.72)	0.3736
19F	3.68(3.36–4.03)	3.56(3.13–4.05)	0.6805	14.68(12.90–16.70)	12.11(10.09–14.54)	1.21(0.97–1.52)	0.0918	76.60(70.65–81.86)	78.63(70.09–85.67)	0.6675
19A	4.18(3.81–4.59)	3.99(3.50–4.56)	0.5861	14.92(13.20–16.87)	13.18(11.08–15.69)	1.13(0.91–1.40)	0.2543	76.17(70.20–81.47)	72.65(63.64–80.48)	0.4724
23F	1.87(1.70–2.05)	1.61(1.41–1.84)	0.0767	7.00(6.24–7.86)	5.73(4.86–6.76)	1.22(1.00–1.49)	0.0509	78.30(72.47–83.39)	75.21(66.38–82.73)	0.5154
1	1.19(1.08–1.32)	1.33(1.15–1.54)	0.2394	8.53(7.36–9.88)	7.68(6.23–9.47)	1.11(0.86–1.43)	0.4225	91.91(87.66–95.06)	88.89(81.75–93.95)	0.3522
2	4.23(3.90–4.60)	4.39(3.90–4.93)	0.626	26.19(23.18–29.60)	21.99(18.49–26.15)	1.19(0.96–1.47)	0.1053	91.91(87.66–95.06)	85.47(77.76–91.30)	0.0601
4	0.71(0.64–0.79)	0.65(0.55–0.76)	0.3333	3.29(2.90–3.73)	2.37(1.98–2.83)	1.39(1.12–1.73)	**0.0032**	82.55(77.08–87.18)	75.21(66.38–82.73)	0.1041
5	0.91(0.84–0.99)	0.93(0.83–1.06)	0.7385	5.75(5.06–6.53)	5.24(4.37–6.27)	1.10(0.88–1.37)	0.4086	92.34(88.16–95.40)	92.31(85.90–96.42)	0.9913
7F	1.70(1.54–1.87)	1.80(1.58–2.06)	0.4646	9.04(7.88–10.36)	7.76(6.39–9.42)	1.16(0.92–1.48)	0.2081	86.38(81.32–90.50)	82.05(73.88–88.53)	0.2844
8	2.93(2.68–3.21)	2.79(2.45–3.17)	0.5351	17.19(15.31–19.29)	12.28(10.43–14.47)	1.40(1.14–1.71)	**0.0011**	95.74(92.31–97.94)	86.32(78.74–91.98)	**0.0015**
9N	1.91(1.71–2.13)	1.91(1.63–2.22)	0.9786	15.42(13.56–17.54)	13.06(10.88–15.68)	1.18(0.94–1.48)	0.1445	94.47(90.73–97.02)	93.16(86.97–97.00)	0.6261
9V	2.14(1.93–2.37)	2.21(1.91–2.55)	0.7264	9.18(8.21–10.27)	7.76(6.62–9.09)	1.18(0.97–1.44)	0.0889	88.09(83.24–91.93)	76.92(68.23–84.21)	**0.0066**
10A	3.34(3.02–3.69)	3.26(2.83–3.76)	0.7949	16.58(14.21–19.34)	11.66(9.37–14.51)	1.42(1.09–1.86)	**0.0101**	80.85(75.23–85.68)	70.94(61.83–78.96)	**0.0358**
11A	2.64(2.37–2.94)	2.57(2.21–2.99)	0.7804	8.93(8.05–9.90)	6.58(5.68–7.61)	1.36(1.13–1.62)	**0.0009**	72.34(66.15–77.96)	58.97(49.50–67.98)	**0.0114**
12F	0.37(0.32–0.43)	0.40(0.32–0.49)	0.5798	2.80(2.45–3.21)	2.73(2.25–3.31)	1.03(0.81–1.30)	0.8287	90.64(86.17–94.04)	91.45(84.84–95.83)	0.8022
15B	4.10(3.69–4.56)	4.04(3.47–4.70)	0.8682	22.34(19.63–25.44)	20.16(16.77–24.22)	1.11(0.89–1.39)	0.3681	88.09(83.24–91.93)	87.18(79.74–92.64)	0.8069
17F	1.86(1.69–2.04)	1.77(1.55–2.02)	0.5767	10.84(9.53–12.33)	8.10(6.75–9.72)	1.34(1.07–1.67)	**0.0108**	90.21(85.68–93.69)	88.03(80.74–93.30)	0.5301
18C	1.99(1.82–2.17)	2.04(1.79–2.31)	0.7597	9.32(8.33–10.44)	7.78(6.63–9.12)	1.20(0.99–1.46)	0.0686	85.11(79.90–89.40)	78.63(70.09–85.67)	0.1281
20	3.96(3.65–4.30)	3.95(3.51–4.44)	0.9645	13.12(11.69–14.72)	12.39(10.53–14.59)	1.06(0.87–1.29)	0.5773	71.49(65.26–77.17)	74.36(65.46–81.98)	0.5703
22F	2.51(2.32–2.72)	2.39(2.14–2.67)	0.4752	8.52(7.66–9.48)	6.84(5.88–7.96)	1.25(1.04–1.50)	**0.0203**	75.32(69.29–80.69)	60.68(51.23–69.59)	**0.0046**
33F	2.70(2.46–2.96)	2.85(2.50–3.25)	0.4929	21.95(19.19–25.11)	18.47(15.26–22.34)	1.19(0.94–1.50)	0.1458	95.32(91.78–97.64)	88.89(81.75–93.95)	**0.0242**

Note: The values in parentheses represent 95% confidence intervals, formatted as (lower bound–upper bound). GMC= geometric mean concentration; GMI= geometric mean fold increase; 95%CI= 95% confidence interval.

Table S3. Serological Response of study participants aged 60+ years to the 23-valent Pneumococcal Polysaccharide Vaccines.

Sero-types	GMC (95%CI) at baseline		*p*-value	GMC (95%CI) post-vaccination		GMC ratio (95%CI) post-vaccination	*p*-value	Seroconversion rate (95%CI)		*p*-value
	Treatment (*n* = 355)	Control (*n* = 178)		Treatment (*n* = 355)	Control (*n* = 178)			Treatment (*n* = 355)	Control (*n* = 178)	
3	0.36(0.33–0.40)	0.39(0.34–0.45)	0.2814	0.87(0.79–0.96)	0.96(0.84–1.09)	0.91(0.77–1.07)	0.2605	58.31(52.99–63.49)	55.06(47.44–62.51)	0.474
6B	2.65(2.44–2.89)	2.79(2.48–3.15)	0.5023	9.52(8.44–10.75)	9.66(8.14–11.46)	0.99(0.80–1.22)	0.8934	73.52(68.61–78.04)	67.98(60.58–74.76)	0.1804
14	8.80(8.15–9.49)	8.30(7.45–9.24)	0.3842	34.64(31.18–38.48)	31.79(27.40–36.88)	1.09(0.91–1.31)	0.3546	73.52(68.61–78.04)	71.35(64.11–77.86)	0.595
19F	4.04(3.74–4.35)	4.18(3.76–4.65)	0.5874	17.80(15.93–19.89)	15.35(13.13–17.96)	1.16(0.96–1.40)	0.1306	81.41(76.96–85.32)	77.53(70.68–83.43)	0.2898
19A	4.51(4.11–4.94)	4.63(4.06–5.27)	0.7556	17.68(15.87–19.70)	15.56(13.35–18.13)	1.14(0.94–1.37)	0.1803	76.34(71.57–80.66)	67.98(60.58–74.76)	**0.039**
23F	2.07(1.91–2.25)	1.94(1.73–2.17)	0.3507	8.78(7.89–9.78)	7.76(6.67–9.04)	1.13(0.94–1.36)	0.1924	82.82(78.48–86.59)	77.53(70.68–83.43)	0.1417
1	1.46(1.36–1.57)	1.48(1.34–1.64)	0.8521	8.31(7.36–9.38)	8.21(6.92–9.74)	1.01(0.82–1.25)	0.9096	87.89(84.03–91.09)	86.52(80.61–91.17)	0.6526
2	5.31(4.92–5.73)	5.47(4.91–6.10)	0.6541	29.99(27.15–33.13)	25.79(22.41–29.68)	1.16(0.98–1.38)	0.0858	90.70(87.19–93.51)	84.83(78.70–89.76)	**0.0431**
4	0.80(0.74–0.87)	0.77(0.69–0.86)	0.6052	3.39(3.06–3.74)	2.47(2.14–2.85)	1.37(1.15–1.63)	**0.0004**	83.10(78.79–86.85)	73.03(65.88–79.40)	**0.0064**
5	0.92(0.86–0.98)	0.97(0.88–1.07)	0.3604	5.16(4.63–5.75)	5.09(4.37–5.94)	1.01(0.84–1.22)	0.8912	89.58(85.92–92.55)	89.89(84.49–93.90)	0.9116
7F	1.92(1.78–2.06)	1.90(1.71–2.10)	0.8719	9.54(8.47–10.75)	9.01(7.62–10.67)	1.06(0.86–1.30)	0.5913	83.66(79.40–87.35)	80.90(74.34–86.39)	0.426
8	2.93(2.73–3.15)	2.98(2.69–3.30)	0.8093	14.44(13.14–15.88)	11.72(10.25–13.39)	1.23(1.05–1.45)	**0.0122**	87.04(83.10–90.35)	79.78(73.12–85.41)	**0.0283**
9N	2.71(2.50–2.93)	2.71(2.42–3.04)	0.9817	18.76(16.88–20.85)	14.41(12.41–16.73)	1.30(1.08–1.56)	**0.0048**	92.39(89.13–94.93)	88.20(82.53–92.55)	0.1108
9V	2.74(2.52–2.98)	2.84(2.52–3.20)	0.6138	12.12(10.97–13.39)	10.98(9.54–12.63)	1.10(0.93–1.31)	0.2594	87.04(83.10–90.35)	82.02(75.58–87.37)	0.122
10A	4.13(3.82–4.46)	3.96(3.55–4.41)	0.5315	18.44(16.36–20.78)	15.29(12.91–18.10)	1.21(0.98–1.48)	0.0763	82.25(77.87–86.09)	74.16(67.07–80.42)	**0.0288**
11A	3.20(2.96–3.46)	3.02(2.71–3.38)	0.4039	8.69(8.00–9.44)	7.52(6.68–8.45)	1.16(1.00–1.33)	**0.0484**	63.66(58.42–68.67)	56.74(49.12–64.13)	0.1218
12F	0.53(0.49–0.58)	0.53(0.47–0.60)	0.9632	3.40(3.02–3.83)	3.47(2.93–4.10)	0.98(0.80–1.20)	0.8452	89.01(85.29–92.07)	88.76(83.18–93.00)	0.9309
15B	5.11(4.70–5.55)	4.73(4.21–5.31)	0.2863	28.27(25.34–31.55)	22.82(19.54–26.64)	1.24(1.03–1.50)	**0.0269**	85.92(81.86–89.36)	80.90(74.34–86.39)	0.1338
17F	2.20(2.03–2.39)	2.37(2.12–2.66)	0.3032	11.94(10.72–13.30)	12.62(10.84–14.70)	0.95(0.78–1.14)	0.5567	88.73(84.97–91.83)	84.27(78.07–89.29)	0.1453
18C	2.30(2.13–2.48)	2.48(2.22–2.77)	0.2596	9.40(8.54–10.35)	8.88(7.76–10.17)	1.06(0.90–1.25)	0.499	83.66(79.40–87.35)	73.60(66.48–79.91)	**0.0059**
20	4.40(4.09–4.73)	4.62(4.17–5.12)	0.4464	16.15(14.49–18.01)	20.68(17.74–24.11)	0.78(0.65–0.94)	**0.0101**	75.49(70.68–79.88)	79.78(73.12–85.41)	0.2684
22F	2.97(2.77–3.19)	2.92(2.64–3.23)	0.7827	8.53(7.79–9.33)	8.19(7.21–9.31)	1.04(0.89–1.22)	0.6142	61.97(56.70–67.04)	60.11(52.52–67.36)	0.6776
33F	3.26(3.00–3.53)	3.37(3.01–3.78)	0.6201	23.74(20.96–26.88)	24.19(20.30–28.84)	0.98(0.79–1.22)	0.8625	92.39(89.13–94.93)	90.45(85.15–94.34)	0.4416

Note: The values in parentheses represent 95% confidence intervals, formatted as (lower bound–upper bound). GMC= geometric mean concentration; GMI= geometric mean fold increase; 95%CI= 95% confidence interval.

Table S4. List of serious adverse events (SAEs).

System Organ Classes/ Preferred Terms	Treatment group (*n* = 1199)	Control group (*n* = 600)
**Total**	6(0.50)	0(0.00)
Infections and infestations	3(0.25)	0(0.00)
Haemorrhagic fever with renal syndrome	1(0.08)	0(0.00)
Tonsillitis	1(0.08)	0(0.00)
Bronchitis	1(0.08)	0(0.00)
Injury, poisoning and procedural complications	2(0.17)	0(0.00)
Joint injury	1(0.08)	0(0.00)
Pelvic fracture	1(0.08)	0(0.00)
Respiratory, thoracic and mediastinal disorders	2(0.17)	0(0.00)
Respiratory failure	1(0.08)	0(0.00)
Asthma	1(0.08)	0(0.00)
Reproductive system and breast disorders	1(0.08)	0(0.00)
Uterine prolapse	1(0.08)	0(0.00)
Skin and subcutaneous tissue disorders	1(0.08)	0(0.00)
Urticaria	1(0.08)	0(0.00)
